# Legal Notes for Institutional Workers

**Published:** 1915-10-09

**Authors:** 


### LEGAL NOTES FOR INSTITUTIONAL WORKERS.

### Parents and the Mental Deficiency Act.

Judging from the decision in *Rex* v. *Radcliffe*, 31 T.L.R. 610, the Courts are to insist on a strict construction of the provisions of the Mental Deficiency Act. Either parent of a mentally defective person may, under Section 2 (1) (*b*) of that statute, present a petition for an order that the defective be sent to an institution; but it has now been laid down that if the petition is presented by the mother while the father is living, his consent in writing must be obtained, unless it is proved that his consent is unreasonably withheld, or that he cannot be found. Then, also, a restrictive interpretation has been put on the phrase "visible means of support" in the clause of the Act which provides that a mentally defective who can be sent to an institution is one that "is found neglected, abandoned, cruelly treated, or without visible means of support." The last-mentioned condition is not satisfied by showing that in fact the person in question has no property, is unable to earn a living, or has no legal claim for maintenance on his parents, but denotes something akin to the other conditions referred to of his being abandoned or neglected. Though a person might have no property, or be unable to earn a living, or without legal right to maintenance, he nevertheless might be in comfortable enough, or comparatively comfortable, circumstances otherwise; so that even before a mentally defective person so poor as the foregoing facts infer can be sent to an institution, it must be shown that he is practically and in fact adrift in the world and destitute, if the application to confine him is made on the ground that he has "no visible means of support."

### Charitable Bequests to Branch Institutions.

The principle stated in the case of *The National Lifeboat Institution*, 31 T.L.R. 340, is one of very great practical interest to all charities, hospitals, or institutions that receive donations from the public. The institution mentioned had power by charter to make by-laws, and they made a rule to the effect that all legacies received by branches of the institution be at once transferred to the main institution. Legacies were left to the Blackpool branch under various forms of bequest. It has been held that with regard to legacies simply left to the Blackpool branch of the National Lifeboat Institution payment of the funds must be made to the central body to be administered by them; the expenditure, however, to be restricted to the area of the branch; but that legacies left to the local committee to be applied in their discretion in connection with the branch, or to be applied by the local committee for relief locally, must not be sent to the central institution, but should be administered by the local body. In determining such a question as the above the intention of the testator is an important element. Usually he wishes to benefit the local branch, of whose operations he has a first-hand knowledge; and the decision—rightly, in our view—does justice to that aspect of such and similar charitable or benevolent bequests.

### Misconduct of Midwives.

While the Mental Deficiency Act is apparently to be placed in the category of Acts to be strictly construed, the Midwives Act of 1902 is of the other class of Acts to which it is thought right to give a liberal interpretation. In *Stock* v. *Central Midwives Board*, 31 T.L.R. 436, it has been held that the misconduct for which under Section 3 of the statute a midwife may be removed from the roll is not limited to misconduct in discharge of her duties as such.

### A Medical Opinion.

One of the points on which an appeal to the Supreme Criminal Court was taken in the "Brides in a Bath" case (*Rex* v. *Smith*, 31 T.L.R. 617) was as to the competency of a question to one of the medical witnesses, who had been permitted to be asked and to answer whether death had been accidental or not. It was argued that while a doctor could rightly give an opinion on a matter of medical science, he had no business with such a question as the above, which was the exact issue for the jury, who had to determine it on facts unbiassed by casual opinions from witnesses. The Court held that the medical witness objected to was only asked for the opinion as to accidental death or not in certain hypothetical circumstances put to him, and that such a question was quite properly admitted. On that point, therefore, as well as on all the other matters of appeal, the case for the accused failed, and sentence of death was subsequently carried out.

